# Acute type A aortic dissection presenting with left main coronary malperfusion

**DOI:** 10.1093/ehjcr/ytag586

**Published:** 2026-08-04

**Authors:** Xiaoxian Liao, Jie Tan

**Affiliations:** Department of Cardiology, The People’s Hospital of Kaizhou District, No. 8, Ankang Road, Hanfeng Street, Kaizhou District, Chongqing 405400, China; Department of Cardiology, The People’s Hospital of Kaizhou District, No. 8, Ankang Road, Hanfeng Street, Kaizhou District, Chongqing 405400, China

## Case presentation

**Figure 1:**
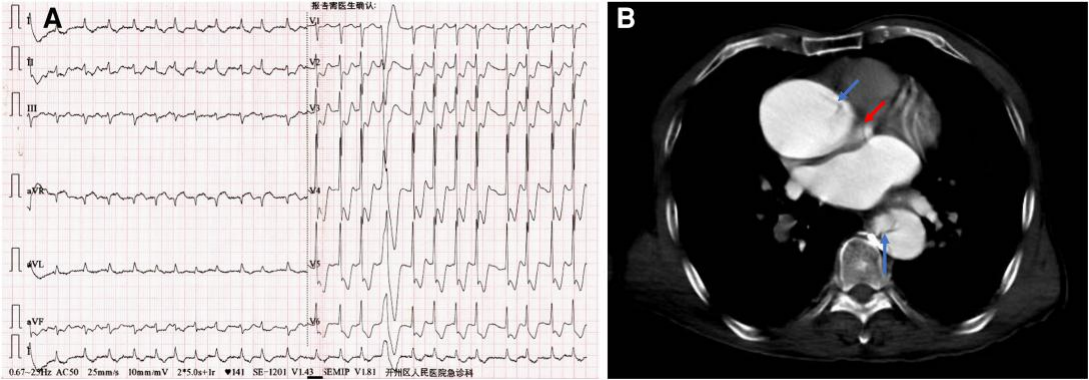
(*A*) Electrocardiogram on admission showing atrial fibrillation with rapid ventricular response, ST-segment elevation in lead aVR, and diffuse ST-segment depression most prominent in leads I, II, aVL, aVF, and V2-V6. This pattern indicates global subendocardial ischaemia and suggests left main coronary artery or multi-vessel ischaemia. (*B*) Computed Tomography Angiography (CTA) showing an intimal flap in the ascending aorta (upper blue arrow) extending towards the left main coronary artery. The proximal left main coronary artery is nearly obstructed by flap-related extension of the dissection (red arrow), explaining the electrocardiographic pattern and haemodynamic collapse.

A 74-year-old man presented with sudden severe chest and back pain for 1 hour. On admission, he was hypotensive (blood pressure 80/58 mmHg), tachycardic (heart rate 141 b.p.m.), and hypoxic (oxygen saturation 90% on room air). Electrocardiography showed atrial fibrillation, ST-segment elevation in lead aVR, and diffuse ST-segment depression in leads I, II, aVL, aVF, and V2-V6 (*Figure 1A*), a pattern consistent with global subendocardial ischaemia and suggestive of left main coronary artery compromise.^1^ Given the abrupt back pain and profound haemodynamic instability, acute aortic dissection was strongly suspected; therefore, bedside echocardiography was not performed, and immediate whole-aorta CTA was prioritized. Emergency contrast-enhanced computed tomography angiography demonstrated acute Stanford type A aortic dissection extending into the left main coronary artery, resulting in near-complete luminal obstruction (*Figure 1B*; blue arrows, aortic dissection; red arrow, left main coronary artery involvement). Coronary malperfusion in type A dissection may result from ostial flap prolapse, extension of the dissection into the coronary artery, or dynamic compression by the false lumen or intramural haematoma; coronary involvement is reported in a minority of patients, and left main coronary artery malperfusion is less frequent than right coronary involvement but can cause extensive myocardial ischaemia and rapid circulatory collapse. Despite vasopressor support and urgent preparation for surgery, the patient developed rapid haemodynamic collapse and died within 1 hour of presentation. This case illustrates the catastrophic consequence of left main coronary malperfusion in acute Stanford type A aortic dissection and highlights the diagnostic value of integrating electrocardiographic findings with immediate aortic imaging.^1,2^

## Data Availability

The data underlying this article are not publicly available due to patient privacy concerns but can be shared on reasonable request to the corresponding author.

## Notes

**Consent:** Written informed consent for publication of the clinical details and images was obtained from the patient’s next of kin. The authors confirm that this consent was obtained in accordance with COPE guidelines.
